# Exploring the potential of a family-based prevention intervention to reduce alcohol use and violence within HIV-affected families in Rwanda

**DOI:** 10.1080/09540121.2016.1176686

**Published:** 2016-07-08

**Authors:** Sumona Chaudhury, Felicity L. Brown, Catherine M. Kirk, Sylvere Mukunzi, Beatha Nyirandagijimana, Josee Mukandanga, Christian Ukundineza, Kalisa Godfrey, Lauren C. Ng, Robert T. Brennan, Theresa S. Betancourt

**Affiliations:** ^a^ Department of Epidemiology, Harvard T.H. Chan School of Public Health, Boston, MA, USA; ^b^ Department of Global Health and Population, Harvard T.H. Chan School of Public Health, Boston, MA, USA; ^c^ FXB-Rwanda, Kigali, Rwanda; ^d^ MPH, Partners In Health/Inshuti Mu Buzima, Kigali, Rwanda; ^e^ ScD, Division of Global Psychiatry, Massachusetts General Hospital, Boston, MA, USA

**Keywords:** Children affected by HIV/AIDS, resilience, IPV, alcohol, Rwanda, family-based prevention

## Abstract

HIV-affected families report higher rates of harmful alcohol use, intimate partner violence (IPV) and family conflict, which can have detrimental effects on children. Few evidence-based interventions exist to address these complex issues in Sub-Saharan Africa. This mixed methods study explores the potential of a family-based intervention to reduce IPV, family conflict and problems related to alcohol use to promote child mental health and family functioning within HIV-affected families in post-genocide Rwanda. A family home-visiting, evidence-based intervention designed to identify and enhance resilience and communication in families to promote mental health in children was adapted and developed for use in this context for families affected by caregiver HIV in Rwanda. The intervention was adapted and developed through a series of pilot study phases prior to being tested in open and randomized controlled trials (RCTs) in Rwanda for families affected by caregiver HIV. Quantitative and qualitative data from the RCT are explored here using a mixed methods approach to integrate findings. Reductions in alcohol use and IPV among caregivers are supported by qualitative reports of improved family functioning, lower levels of violence and problem drinking as well as improved child mental health, among the intervention group. This mixed methods analysis supports the potential of family-based interventions to reduce adverse caregiver behaviors as a major mechanism for improving child well-being. Further studies to examine these mechanisms in well-powered trials are needed to extend the evidence-base on the promise of family-based intervention for use in low- and middle-income countries.

## Introduction

Family-based interventions hold promise for promoting child mental health and well-being for families affected by HIV in low- and middle-income countries (Rochat, Bland, Coovadia, Stein, & Newell, 2011; Rochat, Mkwanazi, & Bland 2013; Rotheram-Borus et al., 2003; Visser et al., 2012). In post-genocide Rwanda, families are often affected by compound stressors, such as HIV, poverty and a legacy of community violence (Betancourt et al., 2014; Russell, Lim, Kim, & Morse, 2015). HIV-affected caregivers experience higher rates of mental-health problems, harmful alcohol use, conflict and intimate partner violence (IPV) (Li et al., 2014; Longmire-Avital, Holder, Golub, & Parsons, 2012; WHO, 2010). HIV-related stressors increase the risk of mental-health concerns and related problems in children, such as anxiety, depression, high-risk sexual behavior, social isolation, stigma, low self-esteem and poor school performance (Betancourt, Meyers-Ohki, Charrow, & Hansen, 2013; Orban et al., 2010). Children exposed to caregiver substance use, distress and IPV are at greater risk of mental-health and developmental problems, and child maltreatment (Bauer, Gilbert, Carroll, & Downs, 2013; Gilbert, Bauer, Carroll, & Downs, 2013; WHO, 2006). Few studies have examined the impact of family intervention on caregiver alcohol use, IPV and child well-being.

The Family Strengthening Intervention for HIV-affected families (FSI-HIV) (Betancourt et al., 2011a) was adapted for use in Rwanda from an evidence-based intervention to promote mental health among children (Beardslee, Gladstone, Wright, & Cooper, 2003). The FSI-HIV seeks to enhance resilience and coping, promote good communication and strengthen relationships within the family. An initial open trial demonstrated that FSI-HIV was highly acceptable and feasible (Betancourt et al., 2014). A subsequent randomized controlled trial (RCT) found that FSI-HIV led to reduced child depression symptoms and improved social support for single caregivers, versus treatment as usual (Betancourt et al., 2014). An exploratory, mixed methods analysis of data generated from the RCT is undertaken here to examine the potential of FSI-HIV to reduce problematic caregiver alcohol use, IPV and family conflict to promote child and family well-being, in HIV-affected families facing multiple stressors in post-genocide Rwanda.

## Methods

A mixed methods approach was used to analyze data collected during the RCT of FSI-HIV versus treatment as usual. This methodology integrates quantitative and qualitative findings in a convergent design (Fetters, Curry, & Creswell, 2013; Guetterman, Fetters, & Creswell, 2015), using data collected pre-intervention, mid-intervention, immediately post-intervention (on average at eight months from baseline) and three months post-intervention. Joint qualitative and quantitative findings strengthen insights into the potential benefits of FSI-HIV (Fetters et al., 2013; Guetterman et al., 2015) that the RCT may have been underpowered to detect. Mixed methods techniques such as joint display (Guetterman et al., 2015) offer exploration of the hypothesis that family-based intervention may prevent adverse caregiver behaviors of problem drinking, family and IPV and attendant effects on child mental health, through strengthened family resilience, improved relationships and family function.

### Study sample

Families affected by caregiver HIV were recruited through referrals from health-center social workers in rural Southern Kayonza District in Rwanda. Inclusion criteria were: at least one adult HIV-positive caregiver living in the household; at least one school-aged child (7–17 years); and caregivers willing to discuss their HIV status with their children. All families enrolled in the study-received treatment as usual through the local health system, consisting of social work support services provided through the HIV clinic. Families were randomly assigned to receive the FSI-HIV family intervention, or to receive the standard-care social work support only. Both single- and dual-caregiver families were eligible to participate, and all children in study households were invited to participate, but could elect not to do so.

### Intervention description

**Figure 1.**
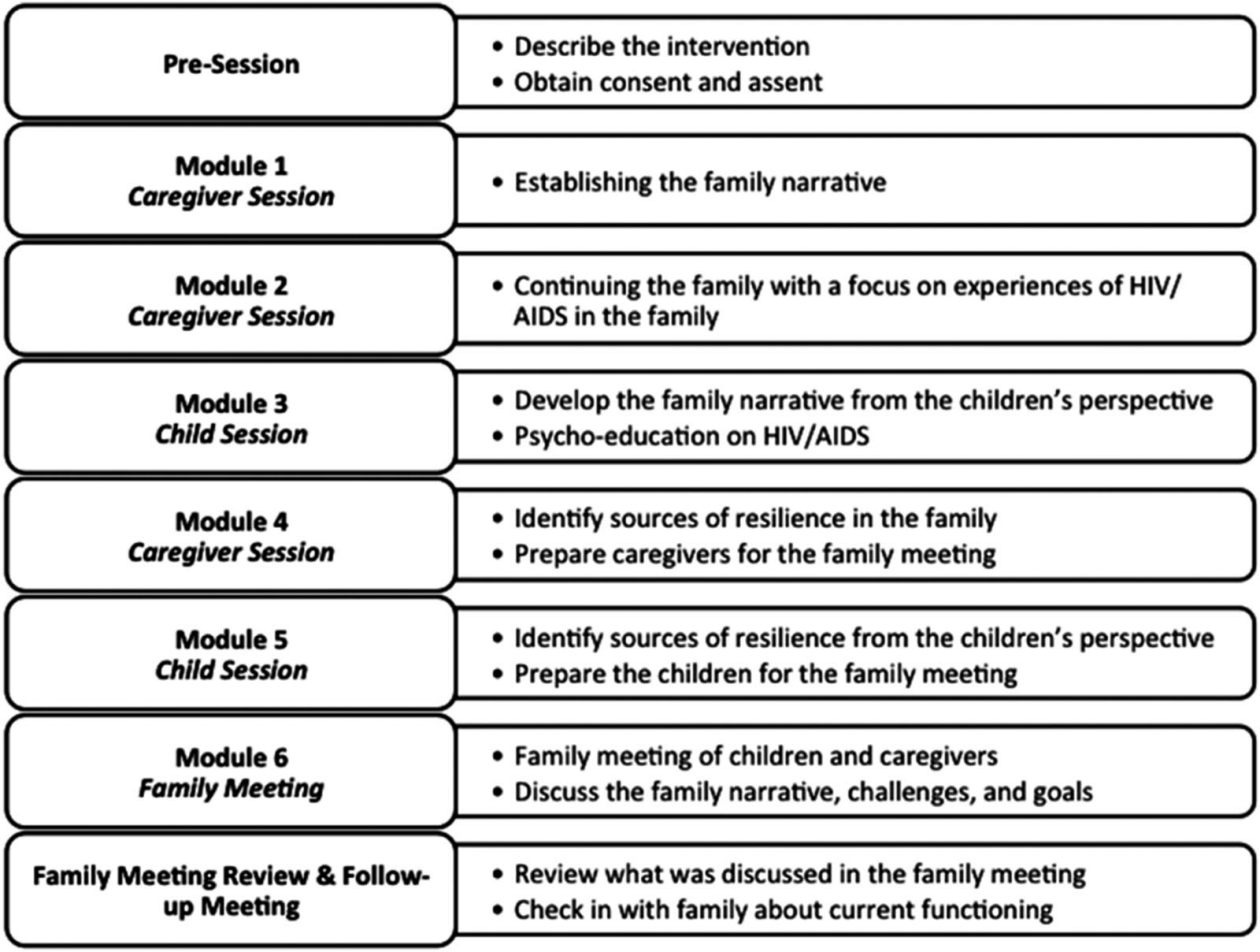
Conceptual model of the FSI-HIV modules.

The FSI-HIV centers on the development of a family narrative to draw upon shared experiences, through four core intervention components: resilience, improved family communication and parenting skills, psycho-education on HIV transmission and status disclosure, and engagement of formal and informal supports (Betancourt et al., 2011a, 2014). Following an introductory meeting, six modules were delivered in families’ homes by trained bachelor-level counselors through a series of interviews with caregivers initially met with counselors during modules 1, 2 and 4. Children undertook separate interviews during modules 3 and 5. Meetings provided opportunity to identify family strengths and challenges, discuss how HIV affects the family and provide strategies to improve communication. This process culminated in a family-led meeting during the final module (module 6; Figure 1), to discuss family challenges and goals for the future (for further details see Betancourt et al., 2014). Modules were completed in a single session of about 90 minutes or during several sessions, averaging 11 sessions over six months with follow-up at three months to check family progress.

**Figure 2.**
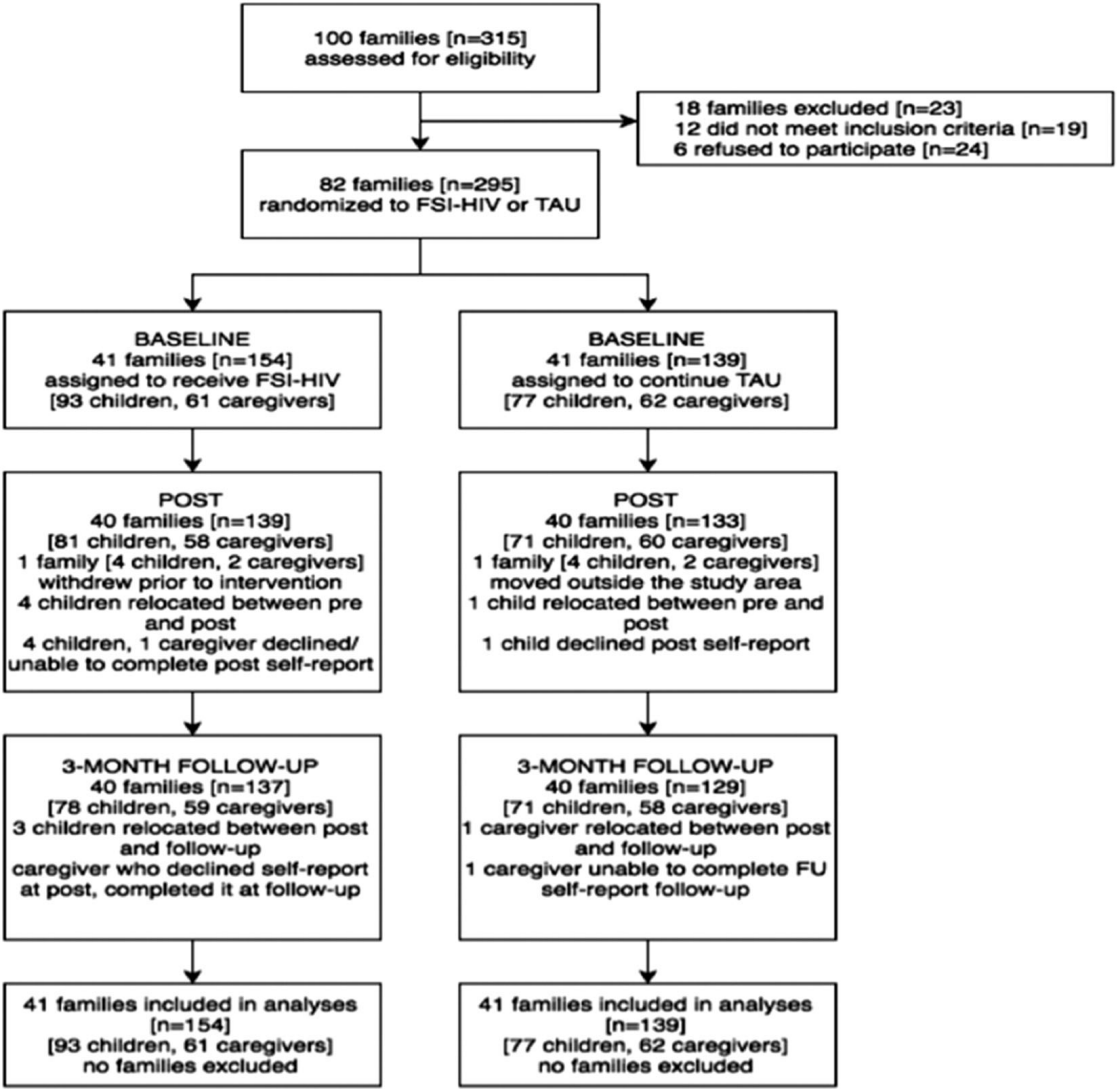
FSI-HIV parent trial study flow.

### Quantitative measures

Questionnaires were administered in families’ homes at baseline, post-intervention and three-month follow-up by local research assistants in Kinyarwanda using smartphones. Questionnaires assessed caregiver and child outcomes. Measures were adapted to fit the local context and underwent a thorough process of forward and back translation (Betancourt et al., 2011c; Van Ommeren et al., 1999).

#### Adapted Alcohol Use Disorders Identification Test (AUDIT; Bohn, Babor, & Kranzler, 1995)

Caregiver alcohol use was assessed using a version of the AUDIT adapted to suit the Rwandan context. AUDIT screens for problematic alcohol use and has been used across diverse settings (Meneses-Gaya, Zuardi, Loureiro, & Crippa, 2009). The total score was the sum of the 11 items (*α* = 0.61 in this sample). Analyses for change in drinking over time were run only for caregivers who reported alcohol use at baseline.

#### Revised conflict tactics scale (Straus, Hamby, Boney-Mccoy, & Sugarman, 1996)

Caregivers who were married or had a partner reported on IPV using an adapted 22-item version of the Conflict Tactics Scale to assess emotional, physical and sexual violence victimization and perpetration. Caregivers reported on the frequency of each form of violence during the past 12 months (0 = not at all, 1 = sometimes, 2 = often). The total score was the sum for all 22 items (*α *= 0.89 in this sample).

#### Child outcomes

Children self-report on mental health and protective processes used measures described in detail in previous publication (Betancourt et al., 2014). Depression was measured using a locally validated version of the Center for Epidemiological Studies Depression Scale for Children (Betancourt et al., 2012; Faulstich, Carey, Ruggiero, Enyart, & Gresham, 1986). Combined anxiety-depression was measured using an adapted Youth Self-Report with a total score of 23 (*α* = 0.93) (Achenbach & Dumenci, 2001). Irritability was measured using a 27-item scale of which 21 were from the Irritability Questionnaire (Craig, Hietanen, Markova, & Berrios, 2008). Functioning was assessed with the 25-item WHO Disability Assessment Schedule for Children validated with Rwandan children (*α* = 0.7) (Scorza et al., 2013). Resilience was measured using an adapted version of the Connor-Davidson Resilience Scale (CD-RISC; Connor & Davidson, 2003) and from local qualitative data (*α* = 0.92). Pro-social behavior was measured using a 20-item scale from local qualitative data (*α* = 0.90) (Betancourt et al., 2011c).

### Qualitative data

Individual semi-structured interviews were completed with all children and caregivers at baseline, post-intervention and follow-up; interviews were audio-recorded for transcription and translation. Additional data were extracted from counselors’ clinical notes to capture observations throughout the intervention (Figure 1). Data analyzed included clinical notes from 35 families, and interview transcripts from 11 families identified as experiencing issues with conflict and alcohol use.

### Analysis

Quantitative analysis was performed using STATA 13.0 and HLM 7.0. Mixed models using Poisson regression assessed differences in change over time in IPV and alcohol use in FSI-HIV versus treatment as usual participants. Models accounted for clustering within families and were adjusted for caregiver sex, age and HIV status. A time variable was included to account for change and a treatment by time interaction term was included to assess the effect of FSI-HIV on outcomes. To better understand the within-couple nature of IPV, we used dyadic modeling (Kenny, Kashy, & Cook, 2006) in a further Poisson model, using multilevel analysis and robust standard errors. Means and standard errors were calculated for outcome scores across three time points and plotted with corresponding confidence intervals as a measure of statistical significance (Figure 3). Figure 3. Graphical representation of mean participant self-reported scores at baseline, post-intervention and at three-month follow-up following a family-based prevention intervention in HIV-affected families in Rwanda. (a) Caregiver self-reports of alcohol and IPV mean caregiver alcohol and IPV scores over baseline, post-intervention and three-month follow-up intervals with 95% confidence intervals and adjusted Poisson regression findings. (b) Child self-reports of mental health and well-being mean child anxiety and depression and depression scores over baseline, post-intervention and three-month follow-up intervals with 95% confidence intervals. (c) Child self-reports of resilience and pro-social behavior mean child resilience and pro-social behavior scores over baseline, post-intervention and three-month follow-up intervals with 95% confidence intervals.

**Figure:**
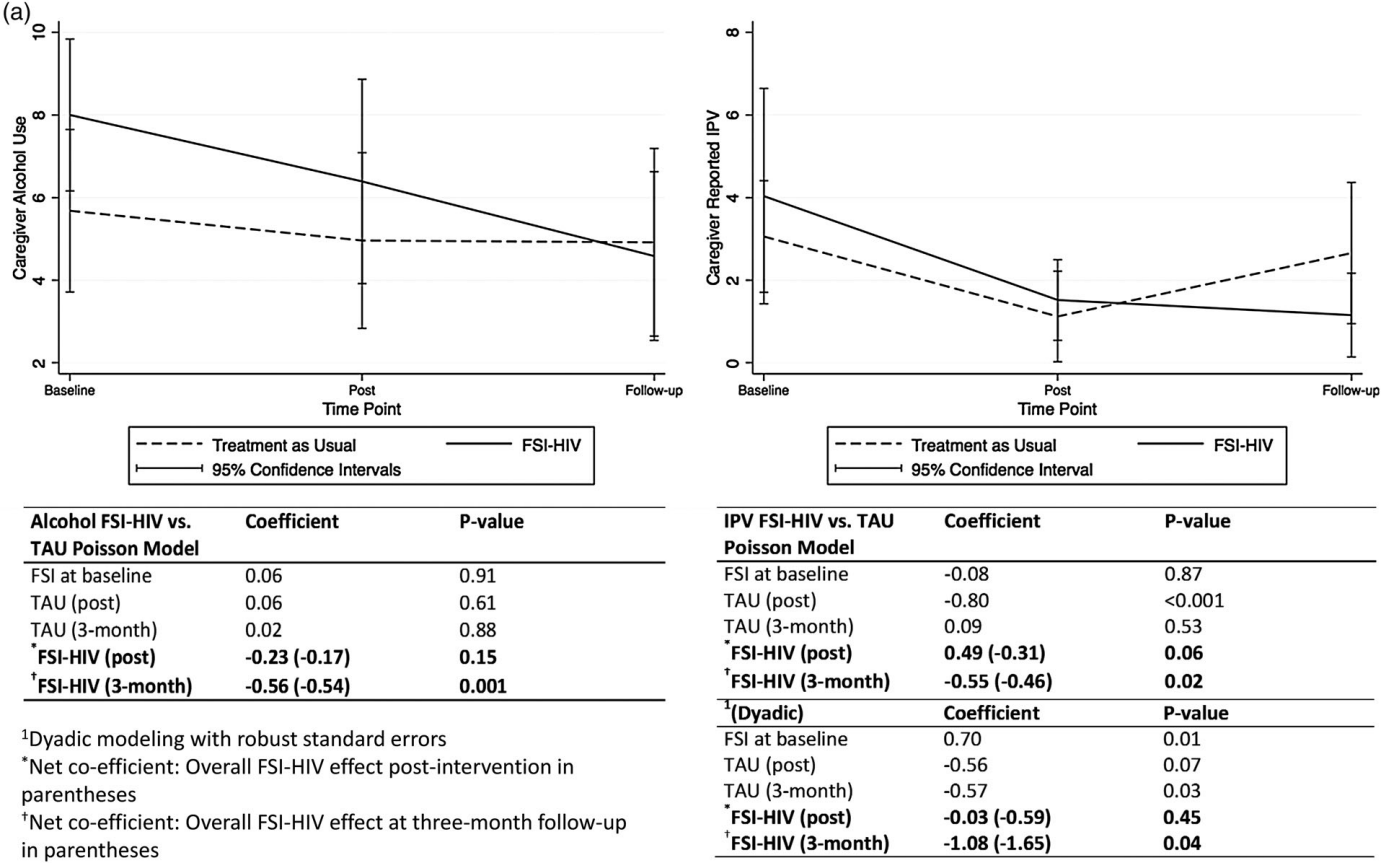

**Figure:**
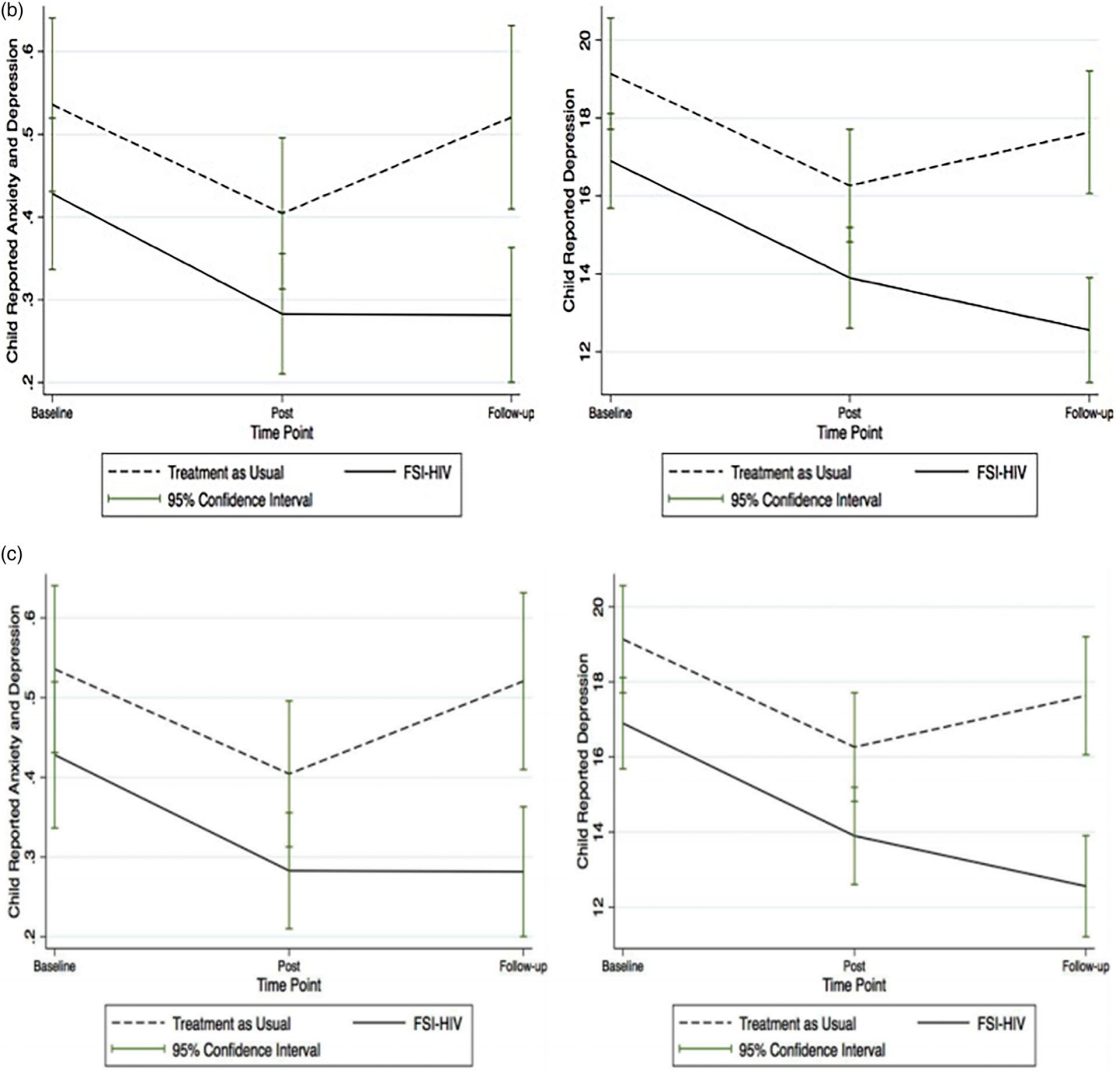


Qualitative data were analyzed using thematic content analysis to identify and analyze patterns driven by a priori research questions (Braun & Clarke, 2006): (1) What, if any, is the relationship between HIV-affected caregiver problem drinking, family and IPV and child mental health? (2) What, if any, impact does the FSI-HIV have on family and IPV and problem drinking and in what way? Data were analyzed inductively to identify codes, which were then further categorized to capture main patterns within the data. Themes from families’ experiences were observed and developed from these categories.

Following quantitative and qualitative data analysis, data were integrated to identify congruence in the findings through a mixed methods convergent design (Creswell, 2015, Creswell & Plano Clark, 2011; Fetters et al., 2013; Guetterman et al., 2015). Additionally, we created a joint display to integrate quantitative and qualitative findings across the progression of the intervention allowing for longitudinal examination of experiences and changes in participants’ scores (Fetters et al., 2013; Guetterman et al., 2015)(Table 3 and Figure 3).

## Results

Forty-one families were randomized to the FSI-HIV and 41 families to treatment as usual, half of each being dual-caregiver families (*n* = 40; 48.9%) so that more caregivers were interviewed overall than children (Figure 2). Of the 61 caregivers in FSI-HIV intervention families, most were female (*n* = 42; 68.9%), HIV-positive (*n* = 52; 85.3%), with a mean age of 41. Of the 93 children interviewed in FSI-HIV families (see Table 1), the majority attended school (*n* = 87, 96.7%) and 6.5% were HIV-positive (*n* = 6).

**Table 1.** Characteristics of family, caregiver and child participants in a RCT of the FSI-HIV.

	Total *n* = 293	FSI-HIV *n* = 154	TAU *n* = 139
Families, no. (%)	82	41 (50)	41 (50)
Dual-caregiver families, no. (%)	40 (48.78)	20 (48.78)	20 (48.78)
Average no. of people per household, mean (SD)	4.86 (1.51)	5.08 (1.46)	4.82 (1.54)
Average no. of children < 18 in household, mean (SD)	3.00 (1.37)	3.17 (1.26)	2.98 (1.36)
SES, mean (SD)	.10 (.08)	.11 (.08)	.10 (.07)
Caregivers, no. (%)	123	61 (49.59)	62 (50.41)
Female, no. (%)	84 (68.29)	42 (68.85)	42 (67.74)
Age, mean (SD)	41.03 (8.76)	41.07 (9.12)	41.00 (8.46)
HIV-positive, no. (%)	103 (83.74)	52 (85.25)	51 (82.26)
Children, no. (%)	170	93 (54.71)	77 (45.29)
Female, no. (%)	83 (48.82)	52 (55.91)	31 (40.26)
Age, mean (SD)	11.76 (2.88)	11.83 (2.84)	11.68 (2.94)
Attends school, no. (%)	151 (93.21)	87 (96.67)	64 (88.89)
HIV-positive, no. (%)	21 (12.35)	6 (6.45)	15 (19.48)

Notes: FSI-HIV family-based intervention adapted for use within Rwandan families affected by HIV. TAU, treatment as usual (usual care or standard of care) comparison group.

### Theme 1: potential of FSI-HIV for reducing alcohol-related problems and family violence in HIV-affected households

Alcohol was reported as a problem for many families in the intervention. Men were more likely to report drinking, and had more harmful drinking than women, at baseline (Table 2). Of the participants reporting alcohol use at baseline, those assigned to FSI-HIV reported less alcohol use over time, compared to treatment as usual participants (Figure 3(a)). Nearly three-fourths (73%) of caregivers married or living with a partner reported an experience of IPV at baseline. Both men and women reported similar rates of emotional violence (50%) perpetration and victimization, and physical violence perpetration (35%). However, significantly more women than men reported physical violence victimization (54% versus 18%, *p* < .03) (Table 2). Graphs indicate reduced IPV among FSI-HIV participants over time compared to treatment as usual (Figure 3(a)). Reductions in alcohol use trend toward significance at the post-intervention time point (*β *= −0.23, *p* = .15) achieving statistically significant reductions (*β *= −0.56 *p* = .01) for the FSI-HIV compared to controls at three months (Figure 3(a)). Reductions in IPV similarly achieve statistically significant reductions in the FSI-HIV compared to controls at three months in Poisson models (*β *= −0.55, *p* = .02) further supported by dyadic modeling results to account for the within-couple nature of IPV (*β* = −1.08, *p* = .04) (see Figure 3(a)). Dyadic model results show an approximate threefold higher reduction in IPV among FSI-HIV participants compared to control treatment as usual participants (net estimates –0.59, –1.65) from the post-intervention to three-month follow-up time intervals (Figure 3(a)).

**Table 2.** Alcohol use and IPV report at baseline.

Alcohol use AUDIT score	Total (*n* = 122)	Women	Men	*P*-value
0 (none)	59%	67%	41%	
1–7 (non-harmful)	25%	23%	31%	
8 or more (harmful)^a^	16%	10%	28%	< .01

Intimate partner violence (IPV)	Total (*n *= 63)	Women	Men	*P*-value
Any IPV	71%	69%	75%	.58
Number of types of IPV (*M*, SD)	3.24 (3.71)	3.86 (4.37)	2.46 (2.52)	.14
Frequency of IPV (*M*, SD)	3.49 (5.33)	4.43 (6.59)	2.32 (2.84)	.12
Any perpetration	62%	57%	68%	.38
Number of types of perpetration (*M*, SD)	1.37 (1.71)	1.57 (1.95)	1.20 (1.49)	.40
Frequency of perpetration (*M*, SD)	1.46 (2.35)	1.43 (2.45)	1.50 (2.27)	.91
Any emotional abuse perpetration	52%	49%	57%	.46
Number of types of emotional abuse perpetration (*M*, SD)	0.63 (.68)	0.63 (0.73)	0.64 (0.62)	.93
Frequency of emotional abuse perpetration (*M*, SD)	0.65 (0.79)	0.66 (0.91)	0.64 (0.62)	.94

^a^Cut-off for harmful drinking reported here for standard AUDIT score and not reflective of the adapted AUDIT score used in this study.

The original RCT was not designed to investigate alcohol and IPV as endpoints. Further investigation in trials specifically powered to detect differences in alcohol and IPV endpoints may further validate this reversal in trends for intervention versus usual-care families demonstrated here at three-month follow-up post-intervention (equivalent to approximately 11 months post-intervention commencement). Within this study, alcohol and IPV reduction appeared to be evident within approximately one year from commencement of the intervention. Further studies are required to better understand the time required between intervention and behavior change.

Many families qualitatively reported one or both caregivers with problematic alcohol use, more frequently among men. Women and children described experiences of alcohol abuse by male caregivers, often related to HIV-related psychosocial stressors, such as accusations of having brought HIV or infidelity into the family, or lack of resources:

The second child is very concerned by her family conflicts where the father is not caring toward the family and beats the mother a lot when he gets drunk. (Counselor, Pre-meeting)

Reports of family violence and alcohol use were highly related to one another. Families described reflection and conscious decision-making to change violent behaviors during the course of the intervention:

The father told the family that he took precautions of no longer fighting because it doesn’t help in any way; the mother also decided to not talk much to the husband when he is drunk because this has been generating conflicts. (Counselor, Module 6)

Throughout the intervention women and children expressed hope that the FSI-HIV may change male caregiver attitudes to alcohol consumption. Meetings provided opportunities to discuss strategies around behavior changes to benefit the family. Toward later stages, caregivers would express commitments to changing behaviors and reducing alcohol intake. Participants described related reductions in intra-family conflict and improved communication and relationships:

The family is doing pretty well; they all noticed family conflicts had reduced. No more fights at home and both parents and children feel more at ease. The wife said: “ if you didn’t come in our family; I might have been murdered by my husband and people could have forgotten about me” (Counselor, Follow-up Meeting)

Joint display (Table 3a and Figure 3(a)) and consideration of integrated quantitative and qualitative findings suggest that FSI-HIV provides an opportunity for problems of excessive alcohol use to be identified, discussed and resolved. Alcohol consumption is both a cause and consequence of HIV infection (Kalichman, Simbayi, Kaufman, Cain, & Jooste, 2007; Fisher, Bang, & Kapiga, 2007) contributing to increased family conflict, as is consistent with prior literature (Kalichman et al., 2007; Li et al., 2014; Russell, Eaton, & Petersen-Williams, 2012). Our findings demonstrate that FSI-HIV facilitated discussion of issues, skill building and development of shared commitment to reverse destructive effects of alcohol on the family. Integrated review of mean scores and qualitative experiences over the course of the intervention highlights the potential for reversal of violent behavior and progression toward improved family functioning (Table 3a and Figure 3(a)).

**Table 3.** Qualitative findings caregiver alcohol use, IPV and child mental-health scores and experiences during the course of an FSI-HIV.

Qualitative findings	Early intervention (pre-sessions or module 1)	Mid-intervention (modules 2–5)	Late intervention (module 6 or family meeting)
	Example quotes		
*3a Theme 1: potential of a family-based intervention for reducing alcohol-related problems and family violence in HIV-affected households*	“The second child is 11 years old and is very concerned by her family conflicts where the father is not caring toward the family and beats the mother a lot when he gets drunk”		“The father told the family that he took precautions of no longer fighting because it doesn’t help in any way; the mother also decided to not talk much to the husband when he was drunk because this has been generating conflicts”
*3b Theme 2: effects of caregiver alcohol use and IPV on child well-being*	“Child is very concerned by her family conflicts where the father is not caring toward the family and beats the mother a lot when he gets drunk. She always feared that the father could hurt them. She’s very close to her mother and reported to have emotional problems as well as her siblings”	“The parents’ behavior traumatized the children because they were often scared”	“The family is doing pretty well; they all noticed family conflicts are reduced. No more fights at home and both parents and children feel more at ease**”**
*3c Theme 3: child and family resilience responses to FSI-HIV*	“Family was divided, parents were not caring about their children, and nothing was going well in the family, children’s school results dropped, there was no good communication” “It has impacted children schooling, parents were no longer working together or take care of their children” “Children dropped out of school”	“The family had a very productive meeting. They reported a change in family conflicts and children are happier because they’re no longer worried and traumatized by their parents fights” “Child is focusing on schooling well and becoming a leader” “When there is a good relationship, children are happy and parents always share with them their CD4 results and when they increase all family members are happy and children think that parents are getting better.”	“They have time to discuss what they’re planning to do and children are also involved in decision making which makes everyone happy” “Parents plan to continue having family meetings to discuss different issues” “Children also are doing well in school and have scored with satisfaction” “The family is doing well; they all noticed family conflicts are reduced. No more fights at home and both parents and children feel more at ease” “The change in the family has been observed even by the community and they’re thankful to FSI because it has helped them so much! The wife said: “if you didn't come in our family, I might have been murdered by my husband and people could have forgotten about me”

### Theme 2: effects of caregiver alcohol use and IPV on child well-being

Post-intervention reductions in depression among FSI-HIV participants were found to be statistically significant in RCT findings (manuscript under review, Betancourt et al., 2014). Child-reported measures of anxiety and depression and depression in Figure 3(b) have non-overlapping 95% confidence intervals when comparing intervention to non-intervention usual-care families. Improvements in child mental health in HIV-affected families experiencing alcohol problems and violence may be clinically significant and merit further investigation in appropriately powered longitudinal studies (Figure 3(b)).

Qualitatively, children described mental-health burdens they attributed to caregiver alcohol use and family violence:

Child is very concerned by her family conflicts where the father is not caring toward the family and beats the mother a lot when he gets drunk   …  She always feared that the father could hurt them   …  She’s very close to her mother and says she has emotional problems. (Counselor, Pre-meeting)

Joint display findings (Table 3b–c and Figure 3(b)–(c)) illuminate the potential FSI-HIV impact on the mechanisms affecting child mental health in families experiencing violence and alcohol-related concerns, through improvements in child resilience and pro-social behavior in family-intervention families.

### Theme 3: child and family resilience responses to family intervention

Graphical display of mean scores in Figure 3(b)–(c) suggests improvements across dimensions of child mental health and child resilience and pro-social behavior. Divergence of the mean scores for the intervention versus usual-care participants suggests that improvements in these measures may be validated in studies powered to detect these differences (Figure 3(c)). Qualitative findings support the suggestion of this mechanism of the family intervention, as being effective in improving child mental health, through the mediator of improved child coping.

Emphasis on identifying sources of resilience within the intervention allowed children the opportunity to reflect on choices they made to improve their coping with their family experiences. Qualitative findings support that they related positive coping to studying, hard work, peer support and performing well at school. Participation in household work and cooperation with their caregivers were described as actions they could take to improve their experiences and well-being. The intervention provided opportunities for families to review goals together. Families set goals of improving communication, reducing conflict and increasing involvement in decision-making. Children expressed relief for opportunity to communicate feelings with caregivers during the intervention, which they described as improving their sense of well-being:

They have time to discuss what they’re planning to do and children are also involved in decision making which makes everyone happy. (Counselor, Follow-up Meeting)

The intervention has helped me to be open, to have someone I can trust and talk to, to socialize with others, has strengthened me, to build hope for the future, setting future goals, being resilient … comforted us and helped us feel like we are not alone. (Mother, post-intervention interview)

Improved school performance in children was also reported over the course of the intervention:

Family was divided, parents were not caring about their children, and nothing was going well in the family, children’s school results dropped, there was no good communication. (Counselor, Pre-meeting)

Child is focusing on schooling well and becoming a leader. (Counselor, Module 3)

Many of the families experiencing alcohol and IPV concerns reported at mid-intervention and late-intervention meetings described benefiting from the opportunities to discuss issues within their relationships and family life. Identifying sources of personal and family resilience built confidence over the course of the intervention in the families’ ability to respond constructively to the alcohol, violence and disease-related stressors they were experiencing:

The family is getting more resilient and open to each other. They have common goals and are working together to achieve them. They’re happy and could probably continue family meeting. (Counselor, Follow-up Meeting)

Integration of quantitative and qualitative findings presented here suggests that child mental health and family functioning improved through the course of the intervention and following the FSI-HIV. Enhanced child resilience was described through improved school performance, relationships with caregivers and supported by improvements in child mental-health scores.

## Discussion

This mixed methods analysis of an RCT of the FSI-HIV captures the experiences of participants and potential positive effects that home-visiting interventions may have on caregiver behavior, family dynamics and child mental health. This study represents an important exploratory investigation of the role of FSI-HIV in interrupting negative trajectories of families affected by HIV, as well as problematic caregiver alcohol use and IPV. An array of approaches to the study of psychological interventions is required to capture relevant effects (Schenk, 2009). The triangulation of data from multiple high-quality sources and informants, for example, caregivers, counselors and children, in this study, combined with integrated quantitative and qualitative data strengthens the potential relevance of these findings (Schenk, 2009; Schenk & Williamson, 2005). Pre- and post-intervention time points are presented here. Further comparison to the experience of families receiving the usual standard of care would strengthen evidence for FSI-HIV impacts (Creswell & Plano Clark, 2011; Fetters et al., 2013). Whilst findings are preliminary in nature, they do indicate the value of further study of family-based prevention for HIV-affected children and their caregivers. Targeted recruitment of caregivers reporting alcohol or IPV-related issues may enable more precise estimation of intervention effects within affected families (Wilson, Graham, & Taft, 2014).

Addressing evidence-based family intervention is complicated by a lack of consensus regarding appropriate measures of psychological health across African studies, rendering comparison of their impacts complex (Sherr, Clucas, Harding, Sibley, & Catalan, 2011). Measures used here were adapted for this context (Betancourt et al., 2011b), whereas alternative measures may yield different results. Well-constructed measures and interventions capture contextual differences better and enhance local sustainability but may limit generalizability (Betancourt et al., 2011b). However, there is a clear need to test and scale up well-tailored evidence-based interventions, especially where preliminary results are promising and early trials demonstrate good acceptability and feasibility (Betancourt et al., 2014; Murray et al., 2011; Patel, Chowdhary, Rahman, & Verdeli, 2011). Improvements in child outcomes may occur as direct effects of the intervention or extraneous factors that are unaccounted for at randomization. Appropriately powered trials and longitudinal studies would enable meditational analyses to further elucidate the complex relationships between caregiver (such as alcohol use and IPV) and child outcomes (such as depression and resilience) and identify specific mechanisms through which the FSI-HIV effects change. This knowledge would enable refinement of the intervention to more specifically target these mechanisms, improving effectiveness and efficiency.

## Conclusion

Expansion of access to care and treatment has proven to fall short of population needs for better integration of mental-health interventions (Freeman, Patel, Collins, & Bertolote, 2005; Wagman et al., 2015). Family-based intervention offers an important opportunity to build cohesion and harness family potential to sustain and support long-term health for children and adolescents growing up in HIV-affected situations of compound adversity (Armistead et al., 2014; Bhana, Mckay, Mellins, Petersen, & Bell, 2010; Biddlecom, Awusabo-Asare, & Bankole, 2009; Doku, 2009; McKernan McKay et al., 2014).

Further studies to investigate effectiveness of family-based intervention in promoting caregiver behavior change and child mental health are warranted. These findings have important implications for further study of interventions to promote long-term child mental health in families experiencing adversity in Sub-Saharan Africa through enhanced family functioning.
